# THE RELATION BETWEEN PROVIDER RESPONSES AND PATIENT AND CARE PARTNER SELF-EFFICACY IN PALLIATIVE CARE APPOINTMENTS

**DOI:** 10.1093/geroni/igac059.2879

**Published:** 2022-12-20

**Authors:** Hannah Silverstein, Meghan McDarby, Brian Carpenter

**Affiliations:** Washington University in St. Louis, St. Louis, Missouri, United States; Memorial Sloan Kettering Cancer Center, New York, New York, United States; Washington University in St. Louis, Saint Louis, Missouri, United States

## Abstract

Person-centered palliative care (PC) fosters patient and care partner participation during medical appointments. The current study used Bandura’s Social Cognitive Theory to examine whether the manner in which healthcare providers respond to patient and care partner questions during initial PC appointments is associated with their self-efficacy in question asking. We analyzed provider responses to direct and embedded questions during initial outpatient PC appointments at a large academic medical center. Provider responses were coded in terms of timeliness (number of dialogue exchanges between a question and a response) and completeness (how fully providers answered questions). Provider responses to direct questions were more timely (M = 1.91, SD = 3.55) compared to responses to embedded questions (M = 4.05, SD = 6.13), t(127) = -3.55, p < 0.001, whereas completeness was similar for both direct (M = 1.43, SD = 0.71) and embedded questions (M = 1.59, SD = 0.96), t(127) = -1.59, p = 0.114. On average, receiving more timely responses was not significantly correlated with self-efficacy, r(49) = -0.038, p = 0.791, nor was receiving more complete responses ρ(49) = -0.178, p = 0.211. These findings suggest that timeliness and completeness of responses may not be factors that affect self-efficacy, thus future research should continue to explore other factors, such as empathy present in provider responses. These findings also suggest that future research should investigate the role and effectiveness of embedded questions as an information-seeking behavior in patients and care partners given that providers took longer to respond to them.

